# Childhood Internalizing and Externalizing Problems Predict the Onset of Clinical Panic Attacks over Adolescence: The TRAILS Study

**DOI:** 10.1371/journal.pone.0051564

**Published:** 2012-12-12

**Authors:** Christina M. Mathyssek, Thomas M. Olino, Frank C. Verhulst, Floor V. A. van Oort

**Affiliations:** 1 Department of Child and Adolescent Psychiatry/Psychology, Erasmus University Medical Center, Rotterdam, The Netherlands; 2 Department of Psychiatry, University of Pittsburgh, Pittsburgh, Pennsylvania, United States of America; Maastricht University Medical Centre, The Netherlands

## Abstract

**Background:**

Panic attacks are a source of individual suffering and are an independent risk factor for later psychopathology. However, much less is known about risk factors for the development of panic attacks, particularly during adolescence when the incidence of panic attacks increases dramatically. We examined whether internalizing and externalizing problems in childhood predict the onset of panic attacks in adolescence.

**Method:**

This study is part of the TRacking Adolescents’ Individual Lives Survey (TRAILS), a Dutch longitudinal population cohort study (N = 1,584). Internalizing and Externalizing Problems were collected using the Youth Self-Report (YSR) and the parent-report Child Behavior Checklist (CBCL) at baseline (age 10–12). At age 18–20, DSM-IV defined panic attacks since baseline were assessed with the Composite International Diagnostic Interview (CIDI). We investigated whether early adolescent Internalizing and Externalizing Problems predicted panic attacks between ages 10–20 years, using survival analysis in univariate and multivariate models.

**Results:**

There were N = 314 (19.8%) cases who experienced at least one DSM-IV defined panic attack during adolescence and N = 18 (1.2%) who developed panic disorder during adolescence. In univariate analyses, CBCL Total Problems, Internalizing Problems and three of the eight syndrome scales predicted panic attack onset, while on the YSR all broad-band problem scales and each narrow-band syndrome scale predicted panic attack onset. In multivariate analyses, CBCL Social Problems (HR 1.19, p<.05), and YSR Thought Problems (HR 1.15, p<.05) and Social Problems (HR 1.26, p<.01) predicted panic attack onset.

**Conclusion:**

Risk indicators of panic attack include the wide range of internalizing and externalizing problems. Yet, when adjusted for co-occurring problem behaviors, Social Problems were the most consistent risk factor for panic attack onsets in adolescence.

## Introduction

The DSM-IV [1] classification includes clinical criteria for both panic attacks and panic disorder. The criteria for a diagnosis of panic attack are a discrete period of intense fear or discomfort, in which four or more out of thirteen (specified) panic symptoms (e.g., palpitations, sweating, trembling or shaking, feeling of choking) developed abruptly and reached a peak within ten minutes. Panic disorder requires (1) recurrent unexpected panic attacks and (2) at least one of the attacks has been followed by at least one month of persistent concern or worry about having panic attacks or its consequences and/or a significant change in behavior related to the attacks. Panic attacks may occur in the context of multiple anxiety disorders. They are considered to be amongst the most debilitating psychiatric conditions [2] and are associated with high level of mental health treatment seeking [3]. While it is known that early identification and subsequent intervention can reduce deleterious outcomes of psychiatric disorders [4], including panic disorder and panic attacks, research on risk factors for the onset of panic attacks is scarce. Some recent studies have identified panic attacks as a risk factor for other anxiety [4]–[6] and mood disorders [4], [5], independent of comorbid internalizing psychopathology. Although there is less consistency, there is some support for panic attacks to precede certain externalizing disorders, including alcohol use disorders [5], [7] and substance use [8], [9]. Hence, identifying predictors for the onset of panic attacks is an important research direction [7], [10].

The reported life-time prevalence of panic attacks when assessed by a clinical interview according to DSM criteria in youth samples ranges from 3.3% [4] to 11.6% [11]. This shows that the reported prevalence rate varies markedly across studies. Importantly, the lower prevalence rate was reported in a sample of 9–17 year olds, and the higher prevalence rate in a sample of 14–16 year olds, indicating that the prevalence rates increase with age in adolescent samples. Even higher lifetime prevalence rates are reported in studies using questionnaires instead of interviews to assess the DSM-criteria (21.4% [5] –63.3% [12]. Females typically have a higher prevalence than males [12], while no differences were found in socio-demographic characteristics between adolescents with and without panic attacks [4]. In addition, a meta-analysis [13] of the heritability of panic disorder revealed that genetic factors accounted for a large proportion of variance (43%). However, to our knowledge, no study has reported heritability for panic attacks.

As the typical age of onset of panic attacks is in late adolescence or early adulthood [14], with a peak between 15 and 19 years [15], it is crucial to examine prospective associations beginning in early adolescence. Identification of predictors of panic attacks early in development is also critical as earlier onsets are associated with increased rates of later psychopathology [16].

The few longitudinal studies on predictors of panic attacks have mostly focused on internalizing problems (emotional problems, e.g. anxiety, depression, other mood disorders). In a sample of high school students assessed over a 4-year period, negative affect [17], [18], anxiety sensitivity [5], [18], as well as separation anxiety disorder [17] were associated with an increased risk for panic attack onset in adolescents. Despite the fact that our knowledge of predictors for adolescent panic attacks is limited, no study to date has prospectively incorporated a broader range of problems, including externalizing problems (behavioral problems, e.g. conduct disorder, oppositional defiant disorder) as possible predictors. Besides internalizing problems, it is important to study other mental health problems as predictors of DSM-IV defined panic attacks since Roza et al. [19] found that both internalizing and externalizing problems in children and adolescents were predictive of anxiety disorders in young adulthood.

The aim of the current study is to extend the limited literature on predictors of panic attack onset, including both internalizing and externalizing problems in early adolescence. We hypothesized that a range of adolescent mental health problems including internalizing problems predict panic attack onset in adolescence. Furthermore, we explore if externalizing problems are part of these predictors. This study is part of the Tracking Adolescents’ Individual Lives Survey (TRAILS), a Dutch general population cohort study following 2,230 children from early adolescence (age 10–12 years) into young adulthood.

## Methods

### Ethics Statement

Written informed consent was obtained at each assessment wave from each participant and their parents. The study was approved by the Dutch Central Medical Ethics Committee (CCMO) and all participants were compensated for their involvement in this study.

### Study Design and Population

Participants were recruited from the general population in five municipalities in the northern part of the Netherlands, including both urban and rural areas. All children living in these municipalities and born between October 1989 and September 1990 (two sites) and October 1990 and September 1991 (three sites) were selected (N = 3,483). Their date of birth and contact information was obtained through the municipality administrations. The first exclusion criterion was non-participation of the school (9.6% of schools, N = 338 children). If the school of a selected child was willing to participate, parents were approached with information brochures (one for themselves and one for their children) and a follow-up phone call in which they were invited to participate. Inability to participate in the study due to severe mental retardation, a severe physical illness, or language-limitations (N = 210) was the second exclusion criterion. Of the 3,483 selected children, 2,935 were eligible for the study, of whom 2,230 (76.0%, of which 50.8% girls) participated in the first wave (T1; 2001–2002; age range 10–12 years). Non-response was due to explicit refusal or inability to establish contact [20]. This response rate was considered adequate given the fact that both parent and child had to agree to participate [21]. Extensive efforts were taken to minimize non-response, including reminder letters and personal house visits [22]. Details of TRAILS have been described elsewhere [21] and are available upon request. Non-response bias was analyzed based on information about mental health determinants and outcomes as reported by teachers of responders and non-responders [20]. Responders and non-responders did not differ in prevalence of psychopathology at T1, and did not differ regarding associations between socio-demographic variables and mental health variables [20]. At T4 (2008–2010; age range 17–20 years), 1,881 respondents (84.3%) continued to participate. Of those, 1,584 subjects provided outcome data, which is 84.2% of T4 participants and 71.0% of T1 participants. When comparing the T1 sample with participants who provided T4 outcome data, we found that T1 predictors of not providing outcome data were male gender (T1 49.2% vs. T4 46.0%), low socio-economic status (T1 25.2% vs. T4 19.6%), ethnic minority background (T1 10.6% vs. T4 7.6%), one-parent family (T1 15.5% vs. T4 13.5%), and a Total Problem score of the Child Behavior Checklist (CBCL/6–18) [23] in the clinical range at baseline (T1 16.1% vs. T4 13.9%).

Of these 1,584 subjects, 405 subjects reported a life-time history of DSM-IV defined panic attacks. Subjects with panic attack onset before the T1 assessment (N = 90) as well as subjects who did not report an age of onset (N = 1) were excluded from the analyses to avoid potential reverse-causal inferences. This rendered N = 1,493 for analyses.

### Instruments

Life-time prevalence of DSM-IV [1] panic attacks and panic disorder was assessed with the Composite International Diagnostic Interview (CIDI) [24], [25]. This is a comprehensive, fully-structured clinical interview for the diagnosis of mental disorders according to the definitions and criteria from DSM-IV. The first part of the CIDI interview consists of a set of probing screening question for all conditions and disorders of interest. If the panic attack/panic disorder screening question is endorsed, the trained interviewer uses the disorder-specific portion of the CIDI to assess exactly which DSM-IV criteria were met as well as the age of onset and frequency of the attacks. A computer algorithm uses these answers to determine whether DSM criteria for disorders were fulfilled. For this study, we used the life-time diagnosis of panic attack and the self-reported age of onset. Good reliability and validity have been reported for the CIDI [26].

For the assessment of internalizing and externalizing problems, we used the Dutch translations of the parent reported Child Behavior Checklist (CBCL/6–18) [23], [27] and the Youth Self-Report (YSR) [28], [29]. Both questionnaires assess internalizing and externalizing problems during the past six months on a 3-point scale (0 = not true; 1 = somewhat/sometimes true; 2 = very/often true). The CBCL and YSR are scored on eight narrow-band syndrome scales: Anxious/Depressed, Somatic Complaints, Withdrawn, Aggressive Behavior, Rule Breaking Behavior, Attention Problems, Social Problems and Thought Problems. In addition, broad-band Internalizing, Externalizing and Total Problem scores can be calculated [23], [28]. The Internalizing Problems score is the sum of the three scales Anxious/Depressed, Somatic Complaints and Withdrawn. Externalizing Problems is the sum of the two scales Aggressive Behavior and Rule Breaking Behavior. The Total Problem score is the sum of the eight subscales as well as additional items not included in the subscales (CBCL: 16 additional items; YSR: 10 additional items). The items and subscales on the youth self-report and the parent-report questionnaire correspond. The Dutch translation has adequate psychometric properties [27], [29].

Socio-economic status (SES) was based on ratings of occupation and education of both mother and father, as well as income. Z-scores of all five components were calculated and categorized into low (lowest 25%), medium (mid 50%) and high (upper 25%) SES. Ethnicity was self-reported and dichotomized as Dutch or non-Dutch.

### Analysis

First, to examine if the panic attack sufferers between T1 and T4 differed from the subjects without panic attacks with respect to sex, age, SES, ethnicity and Internalizing and Externalizing Problems as assessed by their subscale scores of the CBCL and YSR at T1, we used Chi-square tests and analysis of variance (ANOVAs). All descriptive statistics and group comparisons were carried out using SPSS 18 for Windows.

To examine if internalizing and externalizing problems at T1 predict onset of panic attacks over adolescence, we performed continuous time survival analysis, using the maximum likelihood estimator with robust standard error (MLR), which adjusts for non-normality. The baseline hazard was non-parametrical and all survival analyses were carried out in MPlus 5.1 [30]. Sex and SES was included as covariate due to its relation to panic attacks and to multiple dimensions of problem behaviors. Survival time was the interval in years between participants’ age at T1 and age of onset of panic attacks (uncensored), or, in case of no CIDI diagnosis, age at T4 (censored). The resulting hazard ratio of this analysis describes the association between early adolescence internalizing and externalizing problems and onset of panic attacks. They are the log odds of the incremental probability of panic attacks for a unit change in the standardized score (mean = 0; standard deviation = 1) of the predictor. We performed the survival analyses in univariate as well as multivariate models. For all analyses, the significance level was set to *p*<.05.

## Results

Prevalence of panic attacks between T1 (age 10–12) and T4 (age 17–20) was 19.8%, and the prevalence of panic disorder 1.2%. Girls were significantly more likely than boys to have had at least one panic attack between T1 and T4 and individuals from a high SES were significantly less likely to have had at least one panic attack between T1 and T4 than individuals from a low or medium SES background (Table 1). The mean age of onset of panic attacks was 15.8 years (median 16 years) and the age of onset did not differ between girls and boys, F(1) = .837, p = .36.

**Table 1:** Sample characteristics and syndrome sum scores for the unsplit sample, and separately for subjects with and without panic attack onset between T1 and T4.

Variable	Full sample	CIDI Panic attack		
		Yes	No	
	N = 1,493	N = 314 (19.8%)	N = 1,179	
	%/Mean (SD)	%/Mean (SD)	%/Mean (SD)	Significant statistics
Sex				?^2^ (1) = 18.89 ***
Female	53.8%	25.3%	74.7%	
Male	46.2%	16.1%	83.9%	
SES				?^2^ (2) = 7.11 *
Low	19.5%	21.9%	78.1%	
Mid	49.8%	22.9%	77.1%	
High	30.7%	16.6%	83.4%	
Ethnicity				
Dutch	92.6%	20.6%	79.4%	
non-Dutch	7.4%	26.4%	73.6%	
Age at T1	11.1 (.6)	11.1 (.6)	11.1 (.6)	
Panic disorder	1.2%			
CBCL				
Aggressive behavior^1^ (18 items)	5.8 (4.8)	6.0 (4.7)	5.7 (4.8)	
Delinquent behavior^1^ (17 items)	2.0 (2.0)	2.0 (1.8)	2.0 (2.1)	
Anxious/depressed^2^ (13 items)	3.6 (3.1)	4.0 (3.4)	3.4 (3.0)	F (1,1412) = 6.17 *
Somatic complaints^2^ (11 items)	2.0 (2.2)	2.4 (2.3)	1.9 (2.2)	F (1,1408) = 9.64 **
Withdrawn/depressed^2^ (8 items)	1.9 (2.1)	1.9 (2.1)	1.9 (2.1)	
Attention problems (10 items)	4.1 (3.2)	4.0 (3.0)	4.1 (3.3)	
Social problems (11 items)	2.9 (2.8)	3.3 (3.1)	2.8 (2.8)	F (1,1412) = 6.15 *
Thought problems (15 items)	2.2 (2.3)	2.3 (2.3)	2.2 (2.3)	
Externalizing (35 items)	7.7 (6.4)	8.0 (6.1)	7.7 (6.4)	
Internalizing (32 items)	7.5 (5.8)	8.3 (6.2)	7.3 (5.7)	F (1,1412) = 6.33 *
Total (119 items)	27.4 (18.0)	29.2 (18.7)	27.0 (17.8)	
YSR				
Aggressive behavior^1^ (17 items)	5.2 (4.0)	5.9 (4.3)	5.0 (4.0)	F (1,1470) = 11.54 **
Delinquent behavior^1^ (15 items)	3.3 (2.5)	3.8 (2.8)	3.2 (2.4)	F (1,1469) = 12.09 **
Anxious/depressed^2^ (13 items)	4.3 (3.5)	5.1 (3.9)	4.1 (3.4)	F (1,1472) = 18.97 ***
Somatic complaints^2^ (10 items)	4.4 (3.1)	4.9 (3.1)	4.2 (3.0)	F (1,1467) = 10.60 **
Withdrawn/depressed^2^ (8 items)	2.8 (2.3)	3.3 (2.4)	2.6 (2.3)	F (1,1470) = 20.82 ***
Attention problems (9 items)	4.4 (2.7)	4.8 (2.8)	4.3 (2.6)	F (1,1475) = 12.12 ***
Social problems (11 items)	4.1 (3.0)	4.9 (3.4)	3.9 (2.9)	F (1,1473) = 29.07 ***
Thought problems (12 items)	3.4 (3.1)	4.2 (3.5)	3.2 (3.0)	F (1,1470) = 26.86 ***
Externalizing (32 items)	8.5 (6.0)	9.7 (6.6)	8.2 (5.8)	F (1,1470) = 14.01 ***
Internalizing (31 items)	11.4 (7.4)	13.2 (7.9)	10.9 (7.2)	F (1,1471) = 23.53 ***
Total (105 items)	35.9 (19.6)	41.2 (21.2)	34.4 (19.0)	F (1,1460) = 29.55 ***

* <.05 **<.01 ***<.001.

SD = standard deviation; CIDI = Composite International Diagnostic Interview; CBCL = Child Behavior Checklist; YSR = Youth Self-Report; ^1^ part of the Externalizing scale; ^2^ part of the Internalizing scale.

Table 1 shows the sum scores of the CBCL and YSR scales at T1. Adolescents with at least one panic attack between T1 and T4 had higher scores for parent-rated Anxious/Depressed, Social Problems and Internalizing Problems and Somatic Complaints at age 10–12 years than adolescents without a panic attack. Adolescents with at least one panic attack between T1 and T4 had higher scores on all of the self-report YSR scales at T1 than adolescents without a panic attack.

Continuous-time survival analyses were performed to examine whether problems scores at age 10–12 years predicted first onsets of panic attacks during adolescence. The hazard ratios (HR) with 95% confidence interval (CI) adjusted for gender and SES are shown in Table 2 (parent-report) and Table 3 (self-report).

**Table 2:** Results of univariate and multivariate models of survival analysis, predicting the onset of panic attacks with standardized CBCL scores and adjusted for gender and SES.

	Hazard ratio (95% CI)	
CBCL subscales	Univariate	Multivariate^a^
Aggressive behavior^1^	1.09 (0.98–1.22)	1.03 (0.86–1.22)
Delinquent behavior^1^	1.06 (0.95–1.18)	0.98 (0.84–1.15)
Anxious/depressed^2^	1.15 (1.04–1.28)**	1.08 (0.92–1.27)
Somatic complaints^2^	1.15 (1.05–1.26)**	1.11 (1.00–1.23)
Withdrawn/depressed^2^	1.04 (0.93–1.16)	0.90 (0.78–1.04)
Attention problems	1.03 (0.92–1.15)	0.90 (0.78–1.05)
Social problems	1.17 (1.05–1.30)**	1.19 (1.01–1.41)*
Thought problems	1.09 (0.98–1.21)	1.00 (0.86–1.15)
Externalizing	1.09 (0.98–1.21)	1.01 (0.90–1.14)
Internalizing	1.15 (1.04–1.27)**	1.15 (1.02–1.28)*
Total score	1.15 (1.03–1.28)**	

* p<.05 **p<.01 ***p<.0001.

CBCL = Child Behavior Checklist; SES = Socio-economic status; CI = confidence interval;

1 part of the Externalizing scale; ^2^ part of the Internalizing scale; ^a^ the eight subscales were entered into one multivariate model, and the subscales *Externalizing* and *Internalizing* were entered into a separate multivariate model.

**Table 3:** Results of univariate and multivariate models of survival analysis, predicting the onset of panic attacks with standardized YSR scores and adjusted for gender and SES.

	Hazard ratio (95% CI)	
YSR Subscales	Univariate	Multivariate^a^
Aggressive behavior^1^	1.25 (1.13–1.38)***	1.02 (0.87–1.20)
Delinquent behavior^1^	1.26 (1.14–1.40)***	1.11 (0.96–1.29)
Anxious/depressed^2^	1.23 (1.11–1.36)***	0.93 (0.79–1.10)
Somatic complaints^2^	1.16 (1.05–1.29)**	0.97 (0.86–1.10)
Withdrawn/depressed^2^	1.25 (1.13–1.38)***	1.03 (0.89–1.19)
Attention problems	1.21 (1.09–1.36)**	0.95 (0.81–1.11)
Social problems	1.34 (1.21–1.49)***	1.26 (1.06–1.49)**
Thought problems	1.27 (1.16–1.39)***	1.15 (1.01–1.30)*
Externalizing	1.28 (1.16–1.42)***	1.19 (1.04–1.36)**
Internalizing	1.26 (1.14–1.40)***	1.14 (1.01–1.30)*
Total score	1.33 (1.20–1.47)***	

* p<.05 **p<.01 ***p<.0001.

YSR = Youth Self-Report; SES = Socio-economic status; CI = confidence interval;

1 part of the Externalizing scale; ^2^ part of the Internalizing scale; ^a^ the eight subscales were entered into one multivariate model, and the subscales *Externalizing* and *Internalizing* were entered into a separate multivariate model.

The CBCL Anxious/Depressed, Social Problems, Somatic Complaints, Internalizing, and Total Problems scores significantly predicted panic attack onset between T1 and T4 (all *p*s<.01, HR range from 1.15–1.17). As the individual problem scales share variance, we performed multivariate analyses to identify the unique contribution of each CBCL syndrome scale. With all eight syndrome scales in one model, only parent-reported Social Problems (HR = 1.19, 95%CI = 1.01–1.41) at age 10–12 years independently predicted panic attack onset (*p*<.05). In a final model, we examined the broad-band Internalizing and Externalizing problem scores simultaneously. In this model, only Internalizing Problems predicted panic attack onset (HR = 1.15, 95%CI = 1.02–1.28, *p*<.05).

Each of the eight YSR syndrome scales, as well as the Internalizing, Externalizing and Total Problems scales significantly predicted panic attack onset between T1 and T4. When the eight subscales were entered into a multivariate model, only the Social Problems (HR = 1.26, 95%CI = 1.06–1.49, *p*<.01) and Thought Problems (HR = 1.15, 95%CI = 1.01–1.30, *p*<.05) at age 10–12 years scales independently predicted panic attack onset. When examining Internalizing and Externalizing Problems simultaneously, both Internalizing Problems (HR = 1.14, 95%CI = 1.01–1.30, *p<*.05) and Externalizing Problems remained significant (HR = 1.19, 95%CI = 1.04–1.36, *p*<.01).

## Discussion

While having panic attacks increases risk for psychopathology, little is known about predictors of panic attacks. In this study, we measured panic attacks and tested the power of a broad range of both parent- and self-reported problems in 10 to 12 year-old children assessed with the CBCL and YSR to predict the onset of panic attacks across adolescence.

We found a life-time prevalence rate of panic attacks of 19.8%, which is higher than the few previous studies that have assessed panic attacks with a structured interview according to DSM criteria. We suspect that the age range of our study influenced this finding. The other population samples were younger, and lifetime prevalence increases relative to the age of the sample (prevalence 3.3% at age range 9–17 years [4]; prevalence 11.6% at age range 14–16 years [11]; prevalence 19.8% at age range 17–20 years). As the peak onset of panic attacks is between 15 and 19 years, it follows that the prevalence rate of our study sample is higher than in the previous studies.

The parent-reported CBCL scales Anxious/Depressed, Social Problems, Somatic Complaints, Internalizing, and Total Problems scores predicted first onset of panic attack in adolescence. Only the Social Problems and Somatic Complaints syndrome scale predicted panic attack onset when we controlled for shared variance of the scales. All eight self-reported YSR syndrome scales, as well as the Internalizing, Externalizing and Total Problems scales predicted the onset of panic attacks. After controlling for shared variance among the eight syndrome scales and among the Internalizing and Externalizing scales in the multivariate analyses, we found that the Social Problems, Thought Problems and Externalizing scales predicted adolescent panic attack onset.

At first sight, a broad range of problem perceptions are predictive of panic attack onset. This is consistent with Hayward et al.’s suggestion that there are different pathways that lead to adolescent panic attacks [17]; however, their focus was on pathways within the internalizing spectrum of problems. Our findings extend this beyond internalizing problems to include externalizing problems and also a more wide-ranging, general perception of problematic emotions and behavior. All types of problems reported by adolescents on the YSR were risk indicators of later panic attacks. Yet, when we look closer, only a few subscales were independent risk factors. Social Problems was the most consistent risk factor for panic attacks.

This cross-informant consistent result offers broader generalizability and greater theoretical significance than if derived from just one source. The Social Problems scale comprises items such as “acts too young for age,” “too dependent,” “does not get along with other kids,” “gets teased a lot,” “not liked by other kids,” “poorly coordinated or clumsy,” and “prefers being with younger kids”. The long-term consequences of problems with peer relations are supported by findings in other longitudinal research, among which TRAILS: Adolescents who reported to be bullied at age 10–12 years reported persistently higher scores of anxiety symptoms, including symptoms of panic over adolescence, regardless of the continuation of victimization at later ages [31]. In another sample, Roza et al. [19] reported a unique prospective relation between the CBCL Social Problems scale (4–16 years) and lifetime incidence of anxiety disorders (among which panic disorder) assessed 14 years later.

The association found between Social Problems and onset of panic attacks may be the result of a downward spiral starting with poor social skills and difficulties in peer relations, which in turn can lead to even lower self-confidence, and feelings of lack of control and helplessness. Yet, it may also be an expression of genetic transmission of vulnerability from parents to their children, or gene-environment interaction. An adolescent who reports panic attacks is likely to have parents who experience panic attacks or panic disorder [13], [32], [33]. Cross-sectional studies show that adults with panic attacks have problems with relations with others [34]–[36], which may lead to transmission of less developed social skills to their offspring, eventually resulting in social problems in children.

The current study has several strengths and weaknesses. The main strength is that we used a large adolescent sample representative of the general population of adolescents, covering the age range from 10 to 20 years. Also, our predictor variables were assessed prospectively, which avoids selective recall bias. Furthermore, we used information from multiple informants and we assessed panic attacks with strictly defined criteria. The main weakness of this study is that our data were censored at age 17–20 (T4) and consequentially, they did not extend through the entire peak risk period. Generalization of our findings to panic attack onset beyond adolescence will need to be confirmed in future studies. Second, we relied on a snap-shot assessment of clinical symptoms rather than relying on information about longitudinal course of symptoms. It is possible that incorporating symptom course may result in stronger effects. Lastly, our analyses focused on clinical scales to forecast risk for future panic attacks. Additional domains of risk, including social functioning, stress reactivity, anxiety sensitivity [37], and family history could be plausibly related to the onset of panic attacks or panic disorder. Future work in these areas could be interesting. Due to the novelty of our findings, there are limited direct implications for clinical interventions. In the context of prevention, however, there are some potential areas of clinical relevance. First, as associations are found using predictors in early adolescence, early assessment of these indicators can be important for effective preventative intervention. Second, predictors of panic attacks are not limited to the internalizing spectrum only. Assessment of risk for panic attacks is warranted among youth with externalizing problems as well. Thus, by recognizing early risk for panic attacks, problems associated with panic may be averted. Importantly, additional research on predictors of panic attacks is needed before specific clinical intervention recommendations can be formulated.

An important direction for future research is to focus on identifying pathways and mechanisms that explain how early Internalizing, Externalizing and Social Problems increase the risk of panic attack onset in adolescence. In this, an important first step is to identify specific aspects of Social Problems that predict panic attacks. Furthermore, information about panic attacks and panic disorder from first degree relatives should be included to advance understanding of how panic attacks may be transmitted across generations, including genetic and environmental means. Lastly, it is relevant to examine factors that predict and explain the progression from panic attacks to panic disorder. To conclude, we identified childhood Social Problems as consistent independent risk factor of adolescent panic attacks. In addition, we find that the risk markers of developing adolescent panic attacks are diverse and extend beyond problems in the internalizing spectrum to include externalizing problems and a broad-spectrum problems perception.
